# Genomics May Be the Key to Understanding Endurance Training Pillars

**DOI:** 10.3390/genes16030338

**Published:** 2025-03-13

**Authors:** Ricardo Muller Bottura, Daniel Blasioli Dentillo

**Affiliations:** 1 Academy—Health, Science and Performance, São Paulo 01549-020, Brazil; 2 Department of Neuroscience, Rehabilitation, Ophthalmology, Genetics, and Maternal-Infant Sciences (DINOGMI), Università degli Studi di Genova, 16132 Genoa, Italy; 3 Department of Neurosciences, Biomedicine and Movement Sciences, Università degli Studi di Verona, 37124 Verona, Italy; 4 DGLab, Ribeirão Preto 14056-680, Brazil; daniel@dglab.com.br

**Keywords:** genetics, genomics, endurance, VO_2_max, LAn, running economy

## Abstract

Endurance performance is primarily determined by three key physiological pillars: maximal oxygen uptake (VO_2_max), anaerobic threshold, and economy of movement. Recent research has suggested physiological resilience as a potential fourth dimension, referring to an athlete’s ability to sustain performance despite accumulating fatigue. While the role of genetic factors in endurance has been widely studied, their influence on these pillars, particularly on fatigue resistance and long-term adaptation, remains an area of growing interest. This narrative review explores the genomic basis of endurance performance, analyzing genetic contributions to oxygen transport, metabolic efficiency, muscle composition, and recovery. Additionally, it discusses how genetic variability may modulate an athlete’s response to training, including aspects of physiological adaptation, injury susceptibility, sleep, and nutrition. The review highlights physiological resilience in the context of endurance sports, discussing its connection to neuromuscular and metabolic regulation. By integrating genetic insights with established physiological principles, this review provides a comprehensive perspective on endurance adaptation. Future research directions are outlined to enhance our understanding of the genetic underpinnings of endurance, with implications for personalized training and performance optimization.

## 1. Introduction

Traditionally, athletes and coaches recognize the three classic pillars of endurance sports: maximal oxygen consumption (VO_2_max), anaerobic threshold (AT), and economy of movement (EM), which encompasses running economy, cycling economy, and swimming economy [1]. These factors have been widely established in the literature as primary determinants of endurance performance [2,3,4]. Training plans are designed based on tests that assess each of these pillars, allowing for the establishment of training zones that are continuously monitored and adjusted as adaptations occur and athletic performance progresses [5].

However, recent evidence suggests that endurance performance is not solely determined by these three pillars. The concept of physiological resilience has emerged as a fourth key factor, emphasizing an athlete’s ability to sustain performance despite fatigue-induced declines. While the role of genetic factors in endurance has been widely studied, the direct relationship between genomics and the fundamental pillars of endurance training remains underexplored. This review aims to bridge this gap by analyzing how genetic factors influence each of these four pillars and discussing their implications for endurance adaptation and athlete development.

The first pillar of endurance training is VO_2_max. It is directly related to performance and depends on the availability of oxygen, carbohydrates, fats, and mitochondrial density. Additional factors influence or limit VO_2_max improvements. These include capillary density, hemoglobin concentration, stroke volume, aerobic enzyme activity, and muscle fiber type composition [3]. While training and environmental factors play a crucial role in shaping these adaptations, genetic predisposition significantly influences an individual’s baseline VO_2_max and their responsiveness to endurance training.

The second pillar of endurance training is the AT, which represents the maximum intensity at which aerobic metabolism still predominates [4]. Elite athletes tend to have their AT close to their VO_2_max [6]. To achieve this, it is necessary to increase the maximum fat oxidation capacity of skeletal muscle (FatMax) [7,8]. Fat oxidation rates are closely linked to AT, as shown by studies demonstrating a strong correlation between these variables [9].

The third pillar of endurance training is the EM, defined as the amount of energy required to perform a submaximal effort [1]. Various factors influence it, including training type, training cycle phase, strength training, and environmental conditions [2]. However, endurance performance is not only about achieving high physiological efficiency—it is also about sustaining it over time. This ability to resist performance decline despite accumulating fatigue has been increasingly recognized as a key factor in endurance sports. Known as physiological resilience, this concept refers to an athlete’s capacity to tolerate and adapt to unexpected fatigue, playing a crucial role in long-duration events [10].

While training plays a major role in developing this capacity, genetic variability significantly influences an individual’s baseline resilience and ability to adapt to endurance training. Variants in genes related to oxygen transport, metabolic regulation, neuromuscular function, and stress response may modulate an athlete’s ability to sustain prolonged efforts. Despite similar training protocols, genetic variability plays a crucial role in individual adaptations to training [11]. Variants in genes such as *ECA* and *VEGFA* influence oxygen transport efficiency and the response to endurance training. Although multiple factors, such as training, nutrition, and environmental conditions, contribute to endurance performance, in this review, we present evidence supporting the view that genomics may be the key to understanding all pillars of endurance training, as it underlies individual variability in physiological adaptation and performance potential.

## 2. The Genetic Basis of the Three Classical Endurance Pillars

### 2.1. VO_2_max and Its Heritability

Maximal oxygen consumption (VO_2_max) is one of the primary determinants of endurance sports performance, as it sets the upper limit for aerobic energy production and an athlete’s ability to sustain prolonged efforts [3]. VO_2_max represents the highest rate at which the body can absorb, transport, and utilize oxygen during intense exercise [12]. Unlike high-power sports, such as the 100-m sprint, where energy demands are predominantly met by anaerobic metabolism, endurance sports require a coordinated adaptation of multiple physiological systems. To sustain ATP production via aerobic pathways, the body must optimize oxygen uptake, transport, and utilization, which involves adaptations in capillary density, mitochondrial quantity, aerobic enzyme activity, and cardiovascular efficiency [12].

VO_2_max is a central factor in endurance sports performance and is influenced by both physiological adaptations and genetic factors. While training can significantly enhance aerobic capacity, the magnitude of improvement varies among individuals and is largely determined by genetic inheritance. The HERITAGE Family Study demonstrated that up to 47% of the variation in VO_2_max can be attributed to genetic factors, suggesting that polymorphisms in genes related to aerobic adaptation may positively or negatively influence training response [11]. Furthermore, this study highlighted genetic differences in training responsiveness, with some individuals exhibiting substantial increases in VO_2_max, while others show little to no improvement regardless of training load. Since the study was conducted exclusively on sedentary individuals, its findings provide strong evidence that genetic variability influences endurance performance from the very beginning of structured training. This reinforces the importance of understanding genomic contributions to endurance adaptations in both novice and trained individuals.

The primary limiting factor of VO_2_max is oxygen transport capacity rather than muscle oxygen extraction, with cardiac output being one of the most critical determinants of aerobic performance. Classic studies have shown that the increase in VO_2_max between sedentary and trained individuals is strongly associated with differences in maximal stroke volume, whereas maximal heart rate and oxygen extraction by tissues have a lesser influence [13]. This perspective was reinforced by Bassett and Howley [3], who demonstrated that improvements in VO_2_max with training are primarily due to increased cardiac output, driven by a higher stroke volume, while muscle oxygen extraction shows smaller variations. Furthermore, experimental evidence, including interventions that manipulate oxygen delivery (such as blood doping, hypoxia, and β-blocker use), demonstrates that these modifications significantly impact VO_2_max, reinforcing the idea that oxygen delivery to active muscles is the primary limiting factor [3,14].

For oxygen to fulfill its role in aerobic energy production, it must travel through the entire oxygen transport pathway, starting with pulmonary ventilation, passing through the cardiovascular system, and ultimately reaching the mitochondria in muscle cells. This process can be influenced by various physiological factors, including cardiac output, pulmonary diffusion capacity, hemoglobin concentration in the blood, and the distribution of blood flow to active muscles [12].

In addition to central and peripheral adaptations, genetic polymorphisms can influence the efficiency of oxygen transport and, consequently, aerobic capacity. The angiotensin-converting enzyme (ACE) polymorphism has been widely studied in this context, particularly the insertion/deletion (I/D) polymorphism. Individuals carrying the I allele exhibit lower ACE levels, which is associated with better cardiovascular function and greater efficiency in oxygen transport, favoring performance in endurance sports [15]. Conversely, the D allele is linked to higher ACE activity and increased levels of angiotensin II, a potent vasoconstrictor, which may reduce aerobic efficiency. Additionally, polymorphisms in β2-adrenergic receptors can impact primary vasodilation by modulating the response to adrenaline. Individuals with certain variants, such as Gly16Arg and Gln27Glu, may have a lower affinity of the β2 receptor for adrenaline, requiring greater adrenergic activation to promote adequate blood flow [16]. This can lead to an exaggerated compensatory response from the renin–angiotensin system, promoting vasoconstriction and reducing cardiovascular efficiency. When combined with increased angiotensinogen production or higher ACE activity, this scenario may further hinder VO_2_max improvement [11].

The impact of these individual factors helps explain why, even when following identical training programs, athletes show varying degrees of improvement in VO_2_max. The combination of genetic predisposition and the body’s adaptive capacity plays a crucial role in defining the maximum potential for aerobic performance. Studies suggest that the *ACE* I allele occurs more frequently in endurance athletes, whereas the D allele may be more prevalent in sports requiring greater cardiac hypertrophy, such as strength and power disciplines [15].

Another crucial factor for aerobic capacity is angiogenesis, which directly influences VO_2_max and performance in endurance sports. The growth of new muscle capillaries improves oxygen diffusion, enhances blood transit, and reduces dependence on anaerobic glycolysis at higher exercise intensities [3]. Angiogenesis plays an essential role in optimizing oxygen transport and extraction, influencing the diffusion of gases and nutrients in skeletal muscle. Increased muscle capillarization enhances oxygen distribution, prolongs erythrocyte transit time, and reduces diffusion distance—factors that can facilitate the aerobic capacity of endurance athletes [17]. The transport and delivery of oxygen to active muscles are fundamental processes for aerobic capacity and rely on a complex interaction between vascular and genetic mechanisms. Three genes play a crucial role in this context: *VEGFA*, *NOS3*, and *BDKRB2*, which work complementarily to optimize blood flow, promote angiogenesis, and regulate muscle perfusion.

The vascular endothelial growth factor A (*VEGFA*) gene is the primary regulator of angiogenesis, promoting the formation of new blood vessels in response to training and hypoxia, significantly influencing cardiovascular adaptation to exercise. Varillas-Delgado et al. [18] observed that certain genetic variants are associated with higher VO_2_max in elite athletes, indicating an aerobic performance advantage for individuals with favorable genetic profiles. This increase in muscle capillary density allows for greater oxygen delivery to active muscles, optimizing energy production through aerobic metabolism.

Another essential gene in this process is endothelial nitric oxide synthase (*NOS3)*, which is responsible for the production of nitric oxide (NO), a potent vasodilator that regulates blood flow and enhances muscle perfusion during exercise. The study by Ipekoglu et al. [19] found that the *NOS3* rs2070744-T allele is associated with increased NO production, leading to improved blood flow regulation and reduced muscle fatigue during prolonged exercise. The ability to maintain adequate blood flow is crucial for preventing early metabolic acidosis and increasing tolerance to prolonged effort, both fundamental characteristics in endurance events.

Additionally, the Bradykinin B2 receptor (*BDKRB2)* gene plays a fundamental role in the regulation of bradykinin-dependent vasodilation, which is crucial for the efficiency of oxygen transport and muscle blood flow during exercise. Studies have shown that the −9/+9 polymorphism in *BDKRB2* is strongly associated with endurance performance, with elite athletes exhibiting a higher prevalence of the −9/−9 genotype, which is linked to an enhanced vasodilatory response [20]. This increased vasodilation reduces peripheral vascular resistance, promoting better muscle perfusion and more efficient oxygen distribution to active muscles.

The integration of the *VEGFA*, *NOS3*, and *BDKRB2* genes results in a synergistic effect on optimizing aerobic performance. While *VEGFA* enhances muscle capillarization, *NOS3* improves nitric oxide (NO) bioavailability, promotes vasodilation and efficient blood flow, and *BDKRB2* regulates the vasodilatory response to bradykinin. This genetic interaction facilitates improved oxygen delivery and utilization, enabling endurance athletes to sustain intense efforts for prolonged periods [21].

The practical application of these findings in sports science has direct implications for personalizing training strategies, predicting an athlete’s aerobic potential, and adjusting nutritional approaches to optimize NO production and enhance muscle perfusion. Identifying specific genetic variants can provide valuable insights, allowing for more effective interventions to maximize athletic performance [21]. Therefore, the genetics of angiogenesis and vascular regulation play a fundamental role in determining aerobic capacity and performance in endurance sports.

### 2.2. Anaerobic Threshold and Metabolic Adaptations

VO_2_max represents the maximal aerobic capacity and is primarily limited by oxygen transport. However, endurance performance is not solely determined by VO_2_max but also by an athlete’s ability to sustain effort at high intensities. The AT plays a crucial role in this context, as it represents a measure of aerobic power, with its main limiting factor being the muscle’s ability to utilize oxygen for ATP production [4]. Since AT reflects the fraction of VO_2_max that an athlete can sustain over a prolonged period before the excessive accumulation of lactate and metabolic acidosis, it is highly correlated with endurance performance [4,14]. At the AT, lactate production and removal are balanced, allowing exercise to be sustained without an excessive accumulation of fatigue-inducing metabolites [22]. This balance is strongly influenced by the monocarboxylate transporter MCT1, encoded by the *MCT1* gene (rs1049434), which is responsible for lactate uptake in oxidative skeletal muscle for its subsequent utilization as an energy substrate. Individuals with higher MCT1 expression exhibit greater potential to remove and oxidize lactate and H+, delaying their accumulation in the blood and extending the ability to sustain submaximal exercise intensities [23].

Furthermore, the A1470T polymorphism in the *MCT1* gene can affect the rate of lactate and H+ transport, with the A allele being associated with lower blood lactate concentrations during exercise and showing a higher frequency in endurance athletes [24]. This suggests that, while endurance training plays a crucial role in increasing MCT1 expression, optimizing lactate removal and reutilization to sustain the AT for longer periods [23], genetic variants may modulate lactate metabolism efficiency. As a result, these genetic differences can directly influence an athlete’s ability to sustain prolonged aerobic efforts without experiencing premature accumulation of fatigue-inducing metabolites.

The balance between lactate production and removal also plays a key role in promoting fat oxidation at submaximal intensities, reducing the reliance on anaerobic glycolysis. The ability to sustain exercise intensity at the AT is linked to metabolic adaptations in skeletal muscle, including aerobic enzyme activity, glycogen availability, mitochondrial density, cell membrane permeability to fatty acids, and the muscle’s capacity to oxidize them [3,12]. Aerobic enzymes such as citrate synthase and 3-hydroxyacyl-CoA dehydrogenase play a critical role in regulating mitochondrial ATP production, directly influencing the efficiency of energy regeneration through oxidative phosphorylation [4,25].

The availability and efficient mobilization of energy substrates also play a critical role in the AT. Well-trained athletes have a greater capacity to oxidize fats at higher intensities, reducing their dependence on anaerobic glycolysis and enhancing the sustainability of prolonged efforts [9]. This process is regulated by genetic and molecular factors that influence the expression of key genes involved in energy metabolism. Among them are the peroxisome proliferator-activated receptors (PPAR) family of genes, including *PPARA* (rs4253778), *PPARD* (rs2016520), and *PPARGC1A* (rs8192678) [26]. PPARδ, encoded by the *PPARD* gene, is associated with a more oxidative muscle phenotype, optimizing lipid utilization as an energy substrate and increasing endurance capacity [27]. Additionally, muscle glycogen preservation in endurance athletes is directly linked to high PPARδ expression, which allows for greater metabolic efficiency during prolonged exercise [28]. The expression of PPARδ interacts directly with the *PPARGC1A* gene, regulating mitochondrial biogenesis and oxidative metabolism. This interaction significantly influences submaximal effort sustainability and the efficiency of substrate utilization [29]. Endurance athletes have a higher frequency of the C allele of the *PPARD* gene and the G allele of the *PPARGC1A* gene, both of which contribute to enhanced oxidative capacity [26]. Additionally, the GG genotype of the *PPARA* gene is more frequently found in endurance athletes, further reinforcing the role of genetic regulation in lipid metabolism and aerobic training adaptation [30].

The interaction of these genes contributes to increased mitochondrial density, which plays a fundamental role in maintaining the AT. This is because mitochondrial density directly influences the oxidation rate of energy substrates and the muscle’s ability to sustain higher intensities of VO_2_max without significant lactate accumulation [31]. One of the key physiological determinants of AT is the capacity of trained skeletal muscle mitochondria to expand in volume in response to endurance training. This adaptation allows for greater pyruvate oxidation while reducing reliance on carbohydrates as the primary energy source [31]. This mitochondrial adaptation, combined with increased capillary density and improved oxygen diffusion efficiency, enhances the ability to sustain higher exercise intensities before reaching the AT [3,12].

However, for mitochondria to effectively regulate energy metabolism, they require a steady supply of substrates. The activation of the AMPK pathway, in coordination with PPARδ, plays a key role in regulating glucose uptake and adapting metabolism to endurance training, ensuring efficient energy utilization during exercise [32]. Muscle glycogen availability directly influences the duration for which the AT can be sustained, as its depletion can accelerate fatigue and impair performance in long-duration events [7]. Additionally, cell membrane permeability to fatty acids and the muscle’s ability to oxidize them are crucial factors in lipid utilization as an energy substrate at submaximal intensities. A greater reliance on fat oxidation helps preserve glycogen stores, delaying glycogen depletion and metabolic fatigue [7,9].

The relationship between VO_2_max and the AT is fundamental to understanding aerobic performance. While VO_2_max represents the upper limit of an individual’s aerobic capacity, AT determines the highest intensity at which an athlete can perform without an uncontrolled accumulation of lactate [33]. In endurance athletes, improvements in AT tend to be more influential on performance than absolute increases in VO_2_max. This is because the AT reflects muscular and metabolic adaptations that enable the sustained execution of high-intensity, submaximal efforts over extended periods [22].

Based on these factors, we can conclude that the AT is determined by the interaction between mitochondrial capacity, aerobic enzyme activity, and the efficiency of energy substrate utilization, particularly fat oxidation. Endurance training promotes adaptations in these physiological variables, allowing for a delayed accumulation of lactate and enhanced ability to sustain exercise at intensities close to the AT, which is a key factor in endurance performance [3,4,14].

### 2.3. Economy of Movement and Neuromuscular Efficiency

EM can be defined as the energy demand required to sustain a submaximal effort in various endurance sports, such as running, cycling, swimming, and triathlon [2]. This variable represents the relationship between energy cost and maintained velocity, being influenced by biomechanical, physiological, and neuromuscular factors [34]. While training plays a fundamental role in optimizing movement efficiency, genetic factors also contribute by influencing muscle fiber composition, neuromuscular coordination, and metabolic efficiency. Understanding these genetic influences can provide deeper insights into individual variations in movement economy and endurance performance. Improving the EM allows an athlete to use less oxygen to sustain a given intensity, delaying fatigue and optimizing performance [35].

The EM is strongly dependent on the ability of muscle fibers to generate and sustain movement with minimal energy cost. Endurance athletes typically exhibit a higher proportion of type I muscle fibers, which have a high oxidative capacity, greater mitochondrial density, and increased capillarization [36]. However, type II fibers (particularly IIa) also play a crucial role in endurance performance [37]. The ability of these fibers to efficiently meet energy demands during endurance activities can be influenced by genetic expression [38,39], directly impacting EM. Among the genes that influence muscle composition and efficiency, contributing to improved EM, are *ACTN3* (rs1815739) and *COL5A1* (rs12722), while other genes such as *PPARD*, *VEGFA*, and *PPARGC1A* (previously discussed) also play a significant role.

The *ACTN3* gene encodes α-actinin-3, a structural protein found exclusively in type II muscle fibers. However, a polymorphism (R577X) in this gene introduces a stop codon at position 577 of the protein (X allele), preventing its full production [40]. Individuals with the XX genotype have a lower proportion of type II muscle fibers and a greater predominance of oxidative fibers [41], making them more efficient in aerobic energy production. This contributes to better energy efficiency in endurance sports, as these fibers are more resistant to fatigue and have higher mitochondrial density and capillarization [42]. However, the absence of ACTN3 is associated with a greater susceptibility to exercise-induced muscle damage, which may impair recovery in long-duration efforts such as marathons and triathlons [43]. A study involving seventy-one experienced marathon runners found that, despite having similar training volume, weekly mileage, and number of completed marathons, athletes carrying at least one X allele (RX or XX) experienced greater strength loss, higher rate of perceived exertion (RPE), increased muscle soreness, and elevated serum creatine kinase levels compared to RR genotype runners [44].

An increased RPE can be directly associated with a decline in the EM and subsequent performance. Physiological alterations, such as fatigue in the lower limb muscles, can lead to biomechanical changes in running kinematics, increasing the energy cost of movement [45]. This phenomenon may be amplified in XX individuals, who exhibit greater difficulty in muscle recovery and a higher vulnerability to exercise-induced damage, directly affecting their ability to sustain an efficient running pattern over time. Notably, Kenya and Ethiopia, nations that dominate marathon competitions, exhibit an exceptionally high frequency of the RR genotype of the *ACTN3* gene, with over 90% of elite runners carrying at least one functional allele of this variant [46]. This genetic predominance suggests that the presence of α-actinin-3 may confer specific advantages in EM and muscle resilience, enabling these athletes to withstand the extreme physiological demands of a marathon, which requires sustaining approximately 94% of VO_2_max throughout the race, particularly in attempts to break the sub-2-h barrier [47]. While training environment, cultural habits, and early exposure to high-volume running also contribute to endurance success, these factors alone are not sufficient to explain the dominance of East African runners in long-distance events. The genetic predisposition observed in this population likely enhances their ability to adapt to endurance training and optimize movement efficiency, providing an advantage that extends beyond training conditions.

Furthermore, for efficient energy metabolism, with optimized oxygen delivery and high mitochondrial capacity, certain previously discussed genes must be considered. The *PPARD* gene enhances fat oxidation, preserving muscle glycogen stores [27], while *VEGFA* regulates angiogenesis, improving muscle perfusion [26], and *PPARGC1A* promotes mitochondrial biogenesis and the conversion of muscle fibers to a more fatigue-resistant profile [29]. These genetic variants may optimize the EM and endurance performance in elite athletes. These mechanisms are adaptive responses to endurance training, like increased capillarization and higher mitochondrial density in type IIa fibers [48], which further enhance the EM. Thus, RR-genotyped individuals for *ACTN3* may benefit from greater utilization of type IIa fibers during prolonged efforts, ensuring greater metabolic efficiency and lower energy costs during running.

Another muscular factor that may influence the EM is flexibility. In this regard, the *COL5A1* gene (rs12722) has been identified as a relevant marker for long-distance running performance [49,50]. The *COL5A1* gene encodes type V collagen, which plays a crucial role in regulating the structure and stiffness of tendons and ligaments [51]. Individuals with the TT genotype of the BstUI RFLP polymorphism (rs12722) demonstrated better running performance during the Ironman triathlon run segment compared to those carrying the CC genotype [50]. A similar finding was reported in ultramarathon runners (56 km), where TT genotype athletes completed the race in significantly shorter times compared to those with the TC or CC genotypes [49].

This suggests that greater musculotendinous stiffness may optimize the storage and reutilization of elastic energy during running, ultimately improving EM. This characteristic is particularly advantageous in endurance events, where minimizing energy expenditure with each stride can result in superior performance over long distances [35]. Furthermore, the *COL5A1* polymorphism may also influence the incidence of musculoskeletal injuries, a critical factor for longevity and consistency in sports performance [49,50]. Thus, the interaction between *ACTN3* and *COL5A1* appears to be essential for endurance athletes, especially runners, to achieve high performance while reducing injury risk. However, endurance performance is not only about maximizing physiological efficiency but also about sustaining it over time, despite accumulating fatigue.

## 3. Physiological Resilience: The “Fourth Dimension” of Endurance

Historically, endurance sports performance has been explained based on three physiological pillars: VO_2_max, AT, and EM. However, these variables are not static over time and undergo progressive degradation as fatigue accumulates. The ability to resist this deterioration, known as durability [52] or physiological resilience, emerges as a fourth key determinant of performance in long-duration events [10].

The concept of durability represents an athlete’s ability to maintain physiological efficiency and sustain high-intensity efforts over extended periods without a significant decline in critical performance markers [10]. This resilience appears to be directly linked to the interaction between metabolic, neuromuscular, and central fatigue resistance adaptations, being modulated by both genetic factors and training [53]. Individuals with a greater genetic predisposition for the first three pillars may consequently exhibit higher durability, as their physiological systems deteriorate more slowly over time.

In addition to the previously discussed muscular and metabolic adaptations, durability also depends on neural mechanisms and resistance to central fatigue, processes that are, in part, genetically regulated. The *BDNF* gene (rs6265), which encodes brain-derived neurotrophic factor (BDNF), is one of the key regulators of neuroplasticity and may influence an athlete’s ability to sustain high training loads over time. The Val66Met polymorphism results in a substitution of valine for methionine at position 66 of the protein, affecting BDNF secretion and consequently synaptic plasticity [54]. Athletes exposed to high-intensity training demonstrate elevated BDNF levels, suggesting that those capable of tolerating more intense training may develop a more resilient neuromuscular system, better adapted to the demands of prolonged effort [55].

Moreover, endurance training induces a significant increase in BDNF release from the human brain, particularly at rest. This was demonstrated by Seifert et al. [56] in a study involving previously sedentary individuals, where after three months of endurance training, basal BDNF release from the brain increased from 58 ± 106 ng·100 g^−1^·min^−1^ to 206 ± 108 ng·100 g^−1^·min^−1^ (*p* < 0.05). This elevation suggests that regular exercise promotes neuroprotective adaptations and enhances neuronal communication, reinforcing the central nervous system’s resilience to fatigue. The study also showed that in animal models, physical training increases BDNF expression in the hippocampus, a region critical for motor learning and adaptation to the physiological stress of exercise [56]. Thus, BDNF’s influence on durability may be linked to its role in sustaining neuromuscular function over time, reducing perceived fatigue, and optimizing neural efficiency in endurance athletes.

Other genes reported in the review by Semenova et al. [21] may be linked to stress response modulation and mental resilience. The Val158Met polymorphism in the *COMT* gene (rs4680) affects the activity of catechol-O-methyltransferase, an enzyme responsible for dopamine degradation in the prefrontal cortex. Individuals carrying the Val allele (GG) exhibit higher enzymatic activity and lower dopamine levels in this region, which has been associated with greater stress resilience and enhanced performance under high cognitive and physical loads, as observed in combat sports [57]. Given that endurance sports require sustained effort over long periods, the regulation of dopamine levels may also play a role in endurance resilience. Athletes with higher COMT activity may experience reduced mental fatigue and enhanced tolerance to prolonged exertion, potentially influencing their ability to sustain performance under increasing physical and psychological stress. In addition to its role in fatigue regulation, this genetic variant may also benefit endurance athletes by assisting in emotional control and mental endurance under stressful conditions, both of which are essential for high-level performance [58,59].

It is important to emphasize, however, that fatigue resistance is a multifactorial phenomenon, resulting from the interaction of multiple genes and environmental factors, such as training and nutrition. Additionally, further research is needed to fully understand these mechanisms, which could contribute to the personalization of training strategies and the optimization of athletic performance.

## 4. Additional Factors Shaping Endurance Performance

### 4.1. Sleep and Recovery

Sleep is one of the fundamental pillars for physical and mental recovery in endurance athletes, as it regulates essential processes such as muscle protein synthesis, restoration of energy balance [60], and motor memory consolidation [61]. Exposure to irregular sleep schedules and biological clock desynchronization has been associated with decreased athletic performance, increased risk of injuries, and impaired recovery [62].

The circadian cycle or circadian rhythm refers to the regular oscillations of physiological and behavioral processes that occur in an approximately 24-h cycle. These oscillations are regulated by a set of genes known as “circadian clock genes”, which interact through complex networks of transcriptional and post-transcriptional feedback loops [63]. Genetic predisposition to being more active during daytime or nighttime can influence an athlete’s ability to adapt to different training and competition schedules. For instance, morning-oriented athletes may perform better in endurance activities when competing at times that align with their circadian profile [62,64,65]. Similarly, evening-type athletes may face additional challenges related to sleep quality, especially when exposed to training schedules that do not align with their natural biological rhythms. This misalignment can lead to reduced performance, particularly in endurance sports, where recovery and sleep quality are crucial for optimal performance [66,67].

From a molecular perspective, the regulation of the biological clock is known to be linked to the CLOCK-BMAL1 transcription complex, which stimulates the activation of a cascade of genes involved in the body’s circadian control. The *CLOCK* gene (Circadian Locomotor Output Cycles Kaput) plays a central role in regulating the rhythm that influences the sleep–wake cycle [63], with the 3111T/C polymorphism (rs1801260) being the most extensively studied. The presence of the C allele has been associated with sleep disorders and metabolic alterations [68], both of which are critical factors for endurance athletes who rely on adequate recovery and an efficient metabolism to sustain long periods of physical activity. Aligning training sessions and competitions with an athlete’s circadian rhythms can lead to significant performance improvements [69]. Moreover, circadian rhythms influence not only sleep–wake cycles but also metabolic efficiency, hormonal responses, and muscle recovery, all of which are critical for optimizing training adaptations in endurance athletes. Kusumoto et al. [64] highlighted that athletes demonstrate faster times and lower ratings of perceived exertion (RPE) when their training sessions are aligned with their biological rhythms. The interaction between the *CLOCK* polymorphism and these factors may result in significant differences in athletic performance, suggesting that personalizing training regimens based on both an athlete’s genotype and preferred time of activity could be an effective strategy to optimize performance.

The *BDNF* gene, in addition to its central role in cognitive function, also acts as a regulator of physiological processes, including sleep [70]. The Val66Met polymorphism can influence an individual’s vulnerability to sleep deprivation. Met allele carriers have been shown to exhibit lower performance on a neurocognitive test (Stroop Task) compared to Val/Val individuals after 20 h of prolonged wakefulness [71]. Thus, athletes with the Met/Met genotype may experience slower recovery following periods of intense training or long competitions, particularly when exposed to sleep deprivation.

The *COMT* gene is another relevant factor in this context, as the Val158Met polymorphism influences enzyme activity and, consequently, brain dopamine levels—a neurotransmitter that plays a crucial role in sleep regulation and stress response [72,73]. These factors are particularly important for endurance athletes, who frequently face physically and mentally demanding conditions. Adequate dopamine levels are essential for restorative, high-quality sleep [74], and polymorphisms such as Val158Met directly impact these levels. Additionally, the *COMT* gene may influence an individual’s ability to cognitively recover after periods of insufficient sleep. Satterfield et al. [75] demonstrated that *COMT* genotype significantly affects cognitive performance during total sleep deprivation and impairs cognitive control through a frontostriatal dopaminergic mechanism. Individuals carrying the Val allele exhibited greater vulnerability in adaptive decision-making, whereas Met allele carriers showed greater resilience to the cognitive performance deficits induced by total sleep deprivation. Thus, the interaction between *COMT* and *BDNF* variants may be associated with a greater recovery potential [76], as sleep quality is directly linked to muscle health restoration as well as athletic and cognitive performance.

### 4.2. Caffeine

The efficiency of energy utilization and ergogenic aids during long-duration training and competitions depends on an individual’s ability to mobilize ATP from consumed macronutrients. Caffeine is one of the most popular pre-workout ergogenic substances, and its metabolism is regulated by the *CYP1A2* gene (rs762551), which encodes an enzyme of the cytochrome P450 system. Polymorphisms in this gene, such as −163C>A, determine the caffeine metabolic rate, classifying individuals with the A/A genotype as fast metabolizers and those with A/C or C/C genotypes as slow metabolizers [77]. This genetic variation directly influences endurance performance following caffeine ingestion. Fast-metabolizing cyclists (A/A) improved their 10 km time-trial performance after consuming 2 to 4 mg/kg of caffeine, whereas C/C athletes experienced a decline in performance with higher doses [78]. Thus, the ergogenic effect of caffeine is modulated by genetic profile, highlighting the importance of personalized sports supplementation.

Another study on endurance cyclists demonstrated that athletes with the C/C genotype for the serotonin receptor gene *HTR2A* (rs6313) who consumed 4 mg/kg of caffeine before a race achieved an average performance advantage of 1.7 min. However, this positive effect was observed exclusively in fast metabolizers, i.e., those with the A/A genotype of *CYP1A2* [79]. Similarly, young athletes showed a significant ergogenic effect with 6 mg/kg caffeine supplementation, but only if they carried the I/I genotype (84.6% positive response) or I/D genotype (77.5% positive response) of the *ACE* gene (rs4340), compared to D/D carriers. I-allele carriers exhibited improvements in endurance capacity, heart rate, aerobic performance, and perceived exertion. Additionally, endurance capacity was positively correlated with habitual caffeine consumption, but only among I/I genotype athletes [80]. These findings reinforce the influence of genetic factors on ergogenic aid metabolism and their relationship with athletic performance.

### 4.3. Resilience to Training and Overtraining Prevention

The ability to resist overtraining and prevent injuries is essential for athletes exposed to high training volumes. Genetics can influence both the inflammatory response and the tissue repair capacity after training, helping to prevent injuries that could compromise training and competition performance. Interleukin-6 (IL6) plays a key role in the inflammatory response associated with exercise-induced muscle damage, exhibiting both pro-inflammatory and anti-inflammatory effects [81]. Initially, it acts as a mediator of other pro-inflammatory cytokines, such as TNFα, but through a finely tuned regulatory process, it can subsequently suppress inflammation by reducing TNFα production and stimulating the synthesis of anti-inflammatory cytokines [82,83]. Several factors, including exercise duration, intensity, and glycogen availability, influence IL6 production during exercise [82].

The functional polymorphism −174G/C (rs13447445) of the *IL6* gene can affect gene expression and, consequently, circulating IL6 levels. This variant has been associated with lower post-exercise creatine kinase levels, suggesting that a greater IL6 response may be beneficial during intense exercise [83]. The G/G genotype, compared to GC and CC genotypes, has also been reported to exhibit higher IL6 levels in endurance swimmers after 12 weeks of training [84]. Another IL6 polymorphism (rs1800795) has been found to have a higher incidence in athletes with anterior cruciate ligament (ACL) ruptures, while the rs16944 polymorphism of the *IL1-B* gene (interleukin-1 β) appears to have a protective effect against this type of injury, depending on genotype [85]. Additionally, genetic polymorphisms of the tumor necrosis factor α (*TNFA)* have been associated with exercise-induced muscle damage. The −308 G/A polymorphism (rs1800629) influences TNFα expression, with the A allele linked to higher cytokine production, which may contribute to overtraining and modulating creatine kinase response. This same polymorphism has also been associated with patellar and Achilles tendinopathies, providing valuable insights for preventive strategies in athletes [83,86].

Other key genes are involved in protection against oxidative stress and tissue repair. superoxide dismutase 2 (*SOD2)* encodes a mitochondrial enzyme responsible for neutralizing free radicals, converting superoxide anion (NO_2_^−^) into hydrogen peroxide (H_2_O_2_), thereby reducing oxidative stress. The rs4880 (Val16Ala) polymorphism influences antioxidant protection, with the T/T genotype being associated with lower oxidative stress resistance, shorter telomeres (a marker of cellular aging), and elevated C-reactive protein levels after ultra-endurance exercise, suggesting reduced protection against oxidative damage [87]. Beyond the impact of oxidative stress, certain genetic variants may increase an individual’s susceptibility to soft tissue injuries, including muscles, tendons, and ligaments. *COL5A1* is one of the main genes involved in this process, as it encodes a key protein for the formation of type V collagen, which influences the structure and mechanical resistance of connective tissues. The C allele of rs12722 has been associated—though not unanimously—with a lower risk of ligament injuries, including anterior cruciate ligament (ACL) ruptures and chronic Achilles tendinopathy [51,88,89].

Another key gene in extracellular matrix regulation is matrix metallopeptidase 3 (*MMP3),* which encodes a metalloprotease essential for connective tissue remodeling. Variations in this gene can compromise injury recovery, as its enzymatic activity influences the degradation and renewal of the extracellular matrix. The G allele of rs679620 in *MMP3*, in interaction with the T allele of rs12722 in *COL5A1*, has been associated with a higher risk of Achilles tendinopathy [90]. Understanding genetic polymorphisms related to injury susceptibility and oxidative stress can be a strategic factor for training personalization and injury prevention in athletes, minimizing the deleterious effects of genetic predispositions in sports performance. Recent studies suggest that genetic variations directly influence exercise-induced muscle damage and tissue regeneration, reinforcing the need for individualized approaches in sports training [91,92].

## 5. Practical Applications and Ethical Considerations

### 5.1. Personalization of Endurance Training

Genomics has great potential to personalize endurance training by influencing key variables such as VO_2_max, AT, EM, and physiological resilience. The identification of genetic variants can optimize training strategies by adjusting intensity and recovery according to individual predisposition. Genes such as *ACTN3*, *PPARGC1A*, and *VEGFA* are directly linked to muscle fiber composition, mitochondrial biogenesis, and angiogenesis, impacting oxygen transport efficiency and energy metabolism. Additionally, integrating genetic data with nutritional aspects can enhance substrate utilization and extend performance in long-duration events. Despite challenges in practical implementation, combining genetic information with physiological and environmental factors enables more precise decision-making, reducing the risk of injuries and optimizing athletic performance.

### 5.2. Ethical and Practical Limitations

Despite the potential of genomics in sports, its application faces ethical and practical challenges, particularly due to the rise of companies that market simplified and inaccurate genetic tests. Reductionist reports that assign fixed percentages of strength and endurance without considering the complexity of gene–environment interactions promote misinformation and discourage the adoption of genetics as a legitimate tool. Moreover, the lack of preparedness among professionals to correctly interpret these data can lead to errors in practical application and misguided decisions regarding athletic development. From a methodological perspective, genetic research in sports faces difficulties in obtaining homogeneous groups and appropriate controls, making case studies a valuable alternative for deepening the understanding of biological individuality. Nevertheless, the continuation of research is essential to ensure that genetics is used ethically and based on scientific evidence, balancing innovation and responsibility to benefit endurance athletes in a safe and effective manner. Ethical applications of genetic testing in training should focus on individualized adaptation strategies rather than talent identification, ensuring that genetic insights are used to optimize training loads, recovery protocols, and injury prevention strategies. Additionally, education for coaches and sports professionals is crucial to ensure that genetic information is interpreted as a complementary tool, rather than as a deterministic factor in athletic development.

## 6. Conclusions

This review examined the role of genomics in endurance performance, focusing on how genetic factors contribute to VO_2_max, AT, EM, and physiological resilience. While training and environmental factors are essential, genetic variability plays a crucial role in shaping individual adaptation and performance potential. Genes such as *VEGFA*, *NOS3*, and *BDKRB2* regulate oxygen transport and vascularization, while *PPARGC1A* and *PPARD* influence mitochondrial efficiency and metabolic pathways. Additionally, genes like *BDNF* and *COMT* may contribute to neuromuscular endurance and fatigue resistance. Understanding these genetic influences can help refine training approaches, injury prevention strategies, and recovery protocols, allowing for a more personalized and effective approach to endurance performance. With genetic testing becoming more accessible, its integration into sports science should be guided by scientific rigor and practical application. Rather than using genomics solely for talent identification, its true potential lies in optimizing training, recovery, and adaptation strategies for athletes at all levels. Moving forward, well-designed studies with interdisciplinary approaches will be key to bridging the gap between genetic potential and real-world performance in endurance sports.

## Data Availability

No new data were created or analyzed in this study. Data sharing is not applicable to this article.

## Abbreviations

The following abbreviations are used in this manuscript:

VO_2_max	Maximal Oxygen Uptake
AT	Anaerobic Threshold
EM	Economy of Movement
FatMax	Maximum Fat Oxidation
MCT1	Monocarboxylate Transporter 1
BDNF	Brain-Derived Neurotrophic Factor
ACE	Angiotensin-Converting Enzyme
HIF1A	Hypoxia-Inducible Factor 1 α
VEGFA	Vascular Endothelial Growth Factor A
PPARGC1A	Peroxisome Proliferator-Activated Receptor γ Coactivator 1 α
PPARδ	Peroxisome Proliferator-Activated Receptor Delta
MMP3	Matrix Metallopeptidase 3
COL5A1	Collagen Type V α 1
IL6	Interleukin 6
TNFα	Tumor Necrosis Factor α
COMT	Catechol-O-Methyltransferase
CYP1A2	Cytochrome P450 1A2
